# Deskeletonizing the Sigmoid Sinus Is Noncompulsory in Skull Base Surgery: 3D Modeling of the Translabyrinthine Approach

**DOI:** 10.1055/a-2375-7912

**Published:** 2024-08-21

**Authors:** Djenghiz P. S. Samlal, Eduard H. J. Voormolen, Hans G. X. M. Thomeer

**Affiliations:** 1 Department of Otorhinolaryngology and Head & Neck Surgery, Brain Center Rudolf Magnus, University Medical Center Utrecht, Utrecht, The Netherlands; 2 Department of Skull Base Surgery, Brain Center Rudolf Magnus, University Medical Center Utrecht, Utrecht, The Netherlands; 3 Department of Neurosurgery, Brain Center Rudolf Magnus, University Medical Center Utrecht, Utrecht, The Netherlands.

**Keywords:** skull base, cerebellopontine, 3D modeling, anatomy, cadaveric, surgical exposure, sigmoid sinus, sinus thrombosis

## Abstract

**Objectives:**

Sigmoid sinus (SS) compression and injury is associated with postoperative SS occlusion and corresponding morbidity. Leaving the SS skeletonized with a thin boney protection during surgery might be favorable. This study quantifies the effect of the SS position on the operative exposure in the translabyrinthine approach and assesses the feasibility of retracting a skeletonized SS.

**Methods:**

Twelve translabyrinthine approaches were performed on cadaveric heads with varying SS retraction: skeletonized stationary (TL-S), skeletonized posterior retraction (TL-R), and deskeletonized collapsing of the sinus (TL-C). High-definition three-dimensional reconstruction of the resection cavity was obtained. The primary outcome, “surgical freedom” (mm ^2^ ), was the area at the level of the craniotomy from which the internal acoustic porus could be reached in an unobstructed straight line. Secondary outcomes include the “exposure angle,” “angle of attack,” and presigmoid depth.

**Results:**

During TL-R, surgical freedom increased by a mean of 41% (range: 9–92%, standard deviation [SD]: 28) when compared to no retraction (TL-S). Collapsing the SS in TL-C provided a mean increase of 52% (range: 19–95%, SD: 22) compared to TL-S. In most cases, the exposure is the greatest when the SS is collapsed. In 40% of the specimens, the provided exposure while retracting (TL-R) instead of collapsing (TL-S) the sinus is equal or greater than 50% of other specimens in which the sinus is collapsed.

**Conclusion:**

In cases with favorable anatomy, a translabyrinthine resection in which the skeletonized SS is retracted provides comparably sufficient exposure for adequate and safe tumor resection.

## Introduction

During the past decades, an increased attention has been paid to quantify the additional exposure gained through various modified skull base techniques compared to traditional approaches. 1 2 3 4 5 Furthermore, there is a trend in preferences towards minimally invasive and endoscopic skull base surgeries. 6 7 8 9 10 These research lines have clinical relevance owing to their potential to save unnecessary elongation of operative time and associated morbidity. Furthermore, it can aid in selection of patients whose favorable anatomy permits a more reserved bony resection while still granting satisfactory exposure medial to the internal auditory canal (IAC) and superior/inferior aspect of the cerebellopontine angle (CPA). 4 5 An appreciable proportion of this literature has focused on the orbitozygomatic and pterional approaches. 1 2 11 12 13 14 However, quantitative anatomical studies on presigmoid (petrous) approaches remain relatively scarce. 3 4 5 15 16 17 This study, therefore, intents to add to the existing body of knowledge, providing quantitative information describing the anatomical corridor and using some cadaveric specimens with three-dimensional (3D)-reconstruction imaging for optimal measurements. While this study will focus on the translabyrinthine (TL) approach, general lessons are likely transferable to the other presigmoid approaches.

In the TL approach, both prolonged sinus retraction during surgery and pressure caused by fat grafts in the resection cavity after closure can cause compression of the sigmoid sinus (SS), which might increase postoperative morbidity (e.g., cerebrospinal fluid [CFS] leakage, headache, intracranial hypertension, cerebellar infarct). 18 19 20 21 22 Furthermore, SS injury (e.g., during routine deskeletonization) can lead to SS embolization and even pulmonary embolism. 19 If this is the case, an increase in SS-related morbidity is hypothesized when compared to retrosigmoid resection of CPA tumors, throughout which neither the sinus is retracted nor are compressive fat grafts used during closure. Comparative studies on the incidence of SS-related morbidity after CPA tumor resection stratified by presigmoid (e.g., TL) and retrosigmoid approaches remain scarce; results trend toward an increased incidence after presigmoid resection, however in not all studies significancy was reached. 22 23 24 A systematic review and meta-analysis is recommended to further test this hypothesis.

It might be beneficial, with the aim of decreasing SS associated complications, to analyze the feasibility of a TL approach in which the SS remains patent by leaving it skeletonized, instead of ridding it of its bony protection. Several variations of SS skeletonization have been described, including the eggshell method, Bill's island, and total bone removal. During the eggshell method, a thin layer of bone is conserved over the sinus, thus protecting the sinus while sacrificing some mobility. Previously, House and Hitselberger 25 recommended to circumferentially separate the prominent anterior portion of the skeletonized SS with a diamond drill, creating two fragments in the skeletonized bony covering. 26 The created oval shaped bone (Bill's island) could thus be depressed in the SS, increasing mobility and exposure while providing protection to the surface of the sinus during retraction. However, the sharp edge of the bony island increases the risk of sinus wall injury and is therefore not universally preferred over total bone removal. 26 Total bone removal improves sinus mobility, though it sacrifices patency and protection of the sinus. Retracting a fully skeletonized SS posteriorly, instead of merely Bill's island, after retrosigmoid bone removal might increase the mobility of the sinus enough, while protecting against sinus injury and preserving patency.

No previous quantitative analysis was described that compared the effect of SS retraction on the exposure gained by this maneuver during TL resection of CPA tumors. The primary aim is to analyze the additional exposure gained due to retraction of a (de-)skeletonized SS compared to its natural position. It is hypothesized that a posteriorly retracted fully skeletonized SS will provide sufficient exposure for adequate and safe resection of CPA tumors.

## Materials and Methods

### Specimen Preparation and Setting

Six fresh frozen cadaveric heads were used to perform a total of 12 temporal bone dissection on both the right and left sides. Preservation of the specimens was achieved by freezing them to −19 °C, and only locally thawing the area of interest under continuous irrigation during dissection. Jackler's *Atlas of Skull Base Surgery and Neurotology* was used for the dissection steps. 27 All procedures concerned the TL approach with various displacements of the SS: TL-stationary sinus (TL-S), TL-retracted sinus (TL-R), and TL-collapsed sinus (TL-C). Dissection was performed by a single medical student (D.S.) and validated by a senior neurotologist-skull base surgeon (H.T.). Optical magnification (3× to 20 × ) was achieved under an operating microscope (SOM 22 ENT, Karl Kasp GmbH & Co., Asslar/Wetzlar, Germany). After each procedure, the specimen was refrozen. High-resolution cone-beam computed tomography (CT)-scans (scan window 12 × 8 cm; contiguous nonoverlapping slices; NewTom, AllDent B.V., Veenendaal, the Netherlands) were obtained preoperatively and after each procedure.

Raw data from the scans were semi-automatically segmented based on their Hounsfield unit and voxel location and converted to a 3D object with Materialise Mimics 24.0, 3D medical image segmentation software (Materialise NV, Leuven, Belgium). Further manipulation and analysis of this 3D-model took place in Materialise 3-Matic 17.0, Design optimization software (Materialise NV, Leuven, Belgium).

### Surgical Procedures

**Fig. 1:**
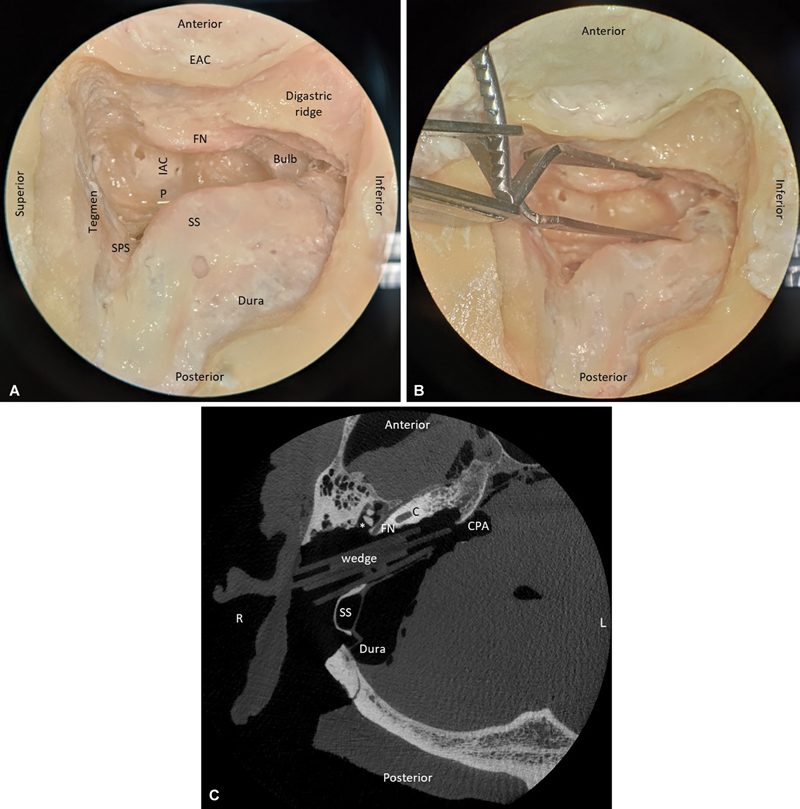
Lateral view of a right-sided translabyrinthine approach without ( **A** ) and with ( **B** ) retraction of the sinus, and transversal postoperative (TL-R) CT-scan with wooden wedges in the resection cavity ( **C** ). Note that the SS remains patent during retraction. * = incus; Bulb, jugular bulb; C, cochlea; CPA, cerebellopontine angle; EAC, external auditory canal; FN, facial nerve; IAC, internal auditory canal; P, porus acusticus internus; SPS, superior petrosal sinus; SS, sigmoid sinus; TL-R, translabyrinthine with retracted patent sinus.

The three procedures were performed progressing sequentially from the least to the most extensive one: TL-S → TL-R → TL-C. The TL approach was performed during the first procedure (TL-S), working between the skeletonized SS and facial nerve. 28 A thin bony covering was conserved over the dura and IAC to improve its radiopacity. To perform dorsal retraction of the skeletonized sinus during the TL-R, up to 20 mm of retro-sigmoid bone was removed. In order to minimize artifacts on the CT-scan, wooden wedges of 1.5 mm in thickness were incrementally positioned in the resection cavity to dorsally mobilize the SS until an additional wedge threatened to decrease the SS patency. Compared to “Bill's island,” the anterior part of the sinus is kept intact, during retraction the mobility arises from the flexing of the thinned bone. Some fracturing of bone may occur in the thicker parts of the eggshell during retraction, especially near the distal portion of the SS. Proximally, a brief transverse part of the sinus may be deskeletonized if retraction causes the eggshell to interfere with the dura. Care was taken to keep the superior petrosal sinus intact. This maneuver is visualized in Fig. 1 .

In order to quantify the last procedure (TL-C), an assumption was made while using the TL-S cadaveric model. To perform the TL-C procedure, the measurements in the 3D model of the TL-S were refitted such that the intersection of the posterior border of the SS and dura was used as the most anteriorly located part of the sinus during TL-C. Thus, this model assumes the walls of a collapsed sinus will lay completely flat along its posterior surface in line with the dura. It was deemed inaccurate to analyze the resection cavity after the retrosigmoid bone removal (TL-R), which would likely result in unbridled amounts of posterior mobilization. Thus, when applying this assumption, the comparability between specimens increases. In doing so, the surgical freedom (SF) in the TL-C is overestimated by the true thickness of the vessel walls. Since this bias does not favor, but disadvantage our hypothesis, the use of it seemed justified. In practice, additional retrosigmoid bone removal is occasionally performed during TL-C to increase exposure. Since, for the sake of comparability, this is not performed in our model, TL-C may not perfectly represent the optimally attainable exposure.

### Exposure Quantification

Different parameters were defined: SF, field of view, and angle of attack. Each provides a quantification of different element that on aggregate can meaningfully inform a surgeon on the expected exposure and ease of resectability.

#### Surgical Freedom

The SF was defined as the area (mm ^2^ ) of the two-dimensional plane at the level of the craniotomy through which surgical instruments can be inserted toward a specific target of interest. This objective quantification technique is based on a refinement of the conical solid method previously used by Schwartz et al, and improved on the accuracy by D'Ambrosio et al in order to quantify irregularly shaped craniotomies. 11 12 In this study, the midpoint on the most proximal IAC surface was used as the primary target point, which forms the tip of the inverted cone. Although not the same, this point (P) projects directly above the internal acoustic porus. Originating from this point (P), six straight lines of sight were placed at various predefined borders of the craniotomy. An oblique plane was generated in the model which intersected both the edges of the craniotomy and the six generated lines. The area on this craniotomy plane which is circumscribed by the six lines is the SF. Any straight instrument inserted through this area can access point P.

**Fig. 2:**
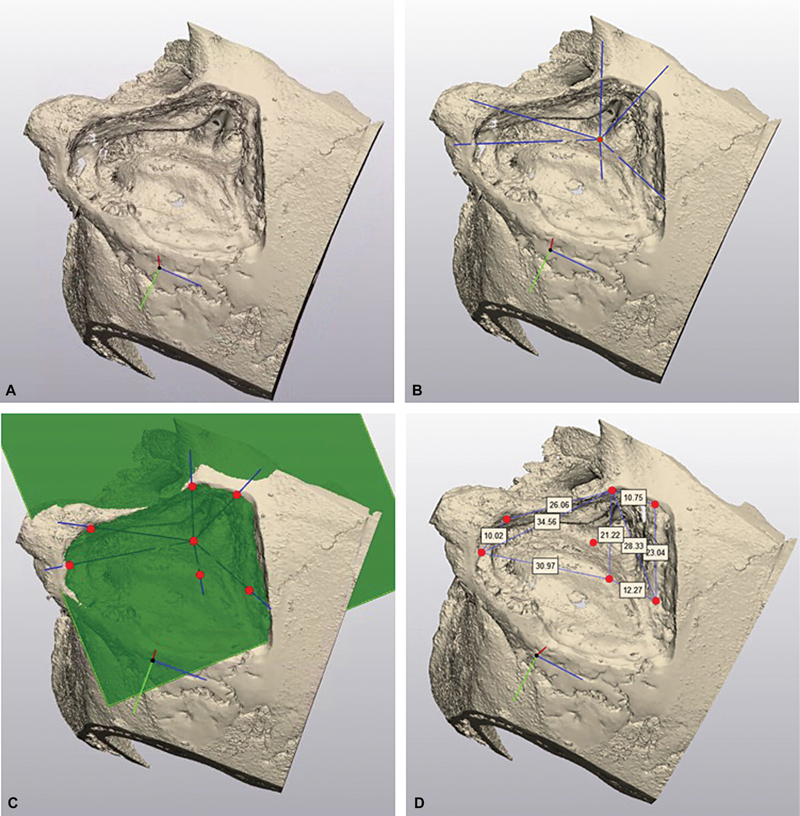
Acquisition of surgical freedom. ( **A** ) Dorsolateral view of a three-dimensional model after left-sided TL-S. ( **B** ) Six lines of sight originating from the porus. ( **C** ) Marking the intersection of these lines with the craniotomy plane. ( **D** ) Measured distances between those intersecting points. TL-S, translabyrinthine without sinus retraction.

A total of six lines were cast from the target point. Line 1 was placed as the most anterior part of the SS directly above (lateral to) the middle of the IAC; Line 2 was placed as far superior-posteriorly until either the sinodural angle or the tegmen was encountered; Line 3 was placed most superior-anteriorly in the craniotomy; Line 4 was placed above the antero-lateral part of the IAC until either the facial nerve or the craniotomy was encountered; Line 5 was placed as far inferior-anteriorly on the craniotomy or until the facial nerve was encountered; Line 6 was placed as far inferior-posteriorly on the craniotomy or until the SS was encountered. The points of intersection between the oblique plane and the six projected lines were identified and four triangles were generated, connecting all points of intersection. 12 Triangles A (points 1, 2, and 4) and B 2 3 4 represent the SF to the superior aspect of the IAC and higher cranial nerves. Triangles C 4 5 6 and D 1 4 6 represent the inferior aspect of the IAC and lower cranial nerves. The areas of these triangles were calculated with Heron's formula. Overall SF was attained by the sum of these triangle areas (mm ^2^ ). See Fig. 2 for the acquisition of SF.

#### Exposure Angle

**Fig. 3:**
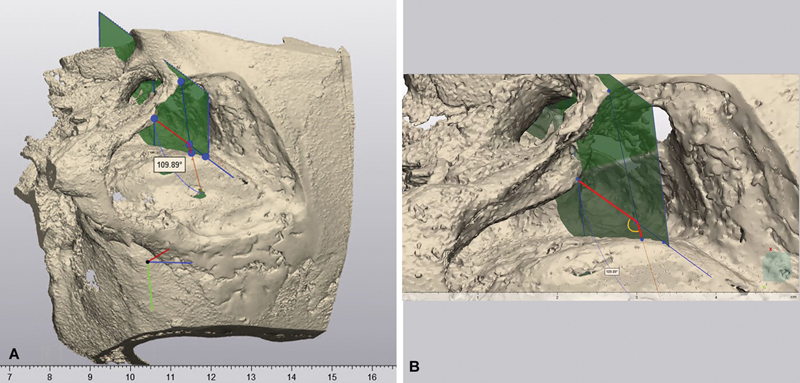
( **A** ) Acquisition of the exposure angle (EA) at the level of the IAC at various magnifications from an inferior-lateral view. ( **B** ) EA is the angle, located by the yellow semicircle, at the intersection between two lines in the transverse plane originating from the craniotomy to the inferior surface of the sigmoid sinus, and originating from the medial part of the facial nerve to the lateral surface of the sigmoid sinus. IAC, internal auditory canal.

Whereas the SF describes the available space to work in with instruments, the “exposure angle” (EA) provides information about the mobility of those instruments. The EA is defined as the angle that a straight instrument could theoretically have in the transverse plane between the SS and the facial nerve within the borders of the craniotomy. For the purpose of this study, the transverse plane is defined as a plane parallel to the surface of the tegmen in order to increase reproducibility between the different CT-scans of the same specimen. To find the EA, the angle between the following two straight lines in this plane is calculated. First, the most horizontal line parallel to the lateral part of the SS and the medial part of the facial nerve is generated. Second, the most horizontal line parallel to the medial part of the SS and the anterior craniotomy border is generated. See Fig. 3A for the acquisition of EA. The steeper this angle is, the less antero-posterior mobility there is. When the EA increases during SS retraction, the space antero-medial to the facial nerve enlarges, resulting in greater accessibility to the distal portion of the IAC. This space is visualized in Fig. 3B .

Since the relative position as well as the absolute distance between the facial nerve, the SS, and CPA varies in both the medial-lateral and the anterior-posterior directions, multiple EA measurements are required to portray the exposure more accurately. The EA is calculated in the transverse plane at the level of the midpoint on the most proximal IAC surface, i.e., point P (EA-IAC). Furthermore, the EA is calculated at the level of the superior portion of the jugular bulb (EA-IAC). The former is chosen to provide information regarding exposure of the IAC. The latter is chosen because the superior portion of the jugular bulb is deemed the most inferior edge of the resection medial to the facial nerve. No EA is calculated superior of the IAC since the facial nerve shortly thereafter passes through the first genu and into the IAC itself.

#### Angle of Attack

**Fig. 4:**
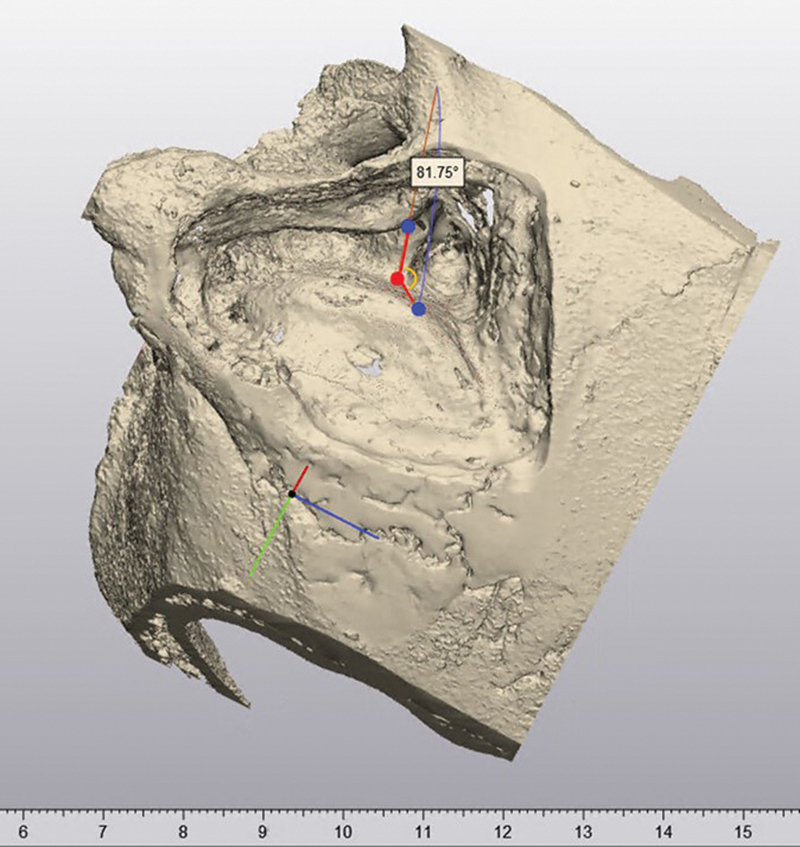
Acquisition of the angle of attack (AA). AA is the angle, located by the yellow semicircle, between the red line over the surface of the IAC and the red line from the sinus to the porus. IAC, internal auditory canal.

The “angle of attack” is defined as the angle between a straight line in the transverse plane from the SS to the midpoint on the proximal IAC surface (point P) and a straight line over the middle of the IAC to its most anterolateral part. A 90-degree angle would entail a straight top-down approach, while a more acute angle (<90 degrees) constrains the approach more anteriorly (overlying SS). An obtuse angle (>90 degrees) allows a more posterior approach in conjunction with an anterior approach. See Fig. 4 for the acquisition of the angle of attack.

### Statistical Analysis

Power analyses were performed to estimate the sample size required to detect a difference in exposure of 15% or greater (α = 0.05, power = 0.9), resulting in a recommended per group sample size of 9 to 10 depending on the specific formulas. Effect size and power were estimated based on previous anatomical skull base studies. 2 3 8 12 The population from which the specimens were randomly gathered is assumed to have a normal distribution of mastoid sizes. Since the different procedures are performed on the same specimens, the variance between the outcomes of those procedures is expected to be homogenic. A random sample of the data did not contain extreme outliers. Thus, a parametric two-tailed paired *t* -test ( *p*_2_ ) was used to determine statistical significance in the cases which compared TL-R and TL-C. However, due to the nature of the maneuver and clinical experience, any amount of SS retraction will increase the exposure when compared to TL-S; therefore, a one-tailed paired *t* -test ( *p*_1_ ) is preferred to determine statistical significance in cases which compared either TL-R or TL-C to TL-S. In all cases, *p* <0.05 was considered significant. Microsoft Excel 2016 was used for data management. All statistical tests were performed in SPSS v28.

## Results

### Specimen Preparation and Characteristics

In all 12 temporal bones, a classical TL dissection was achieved with exposure of the IAC. There were two cases (16.7%) of a high riding jugular bulb in which the superior aspect of the bulb projected over or touched the inferior border of the IAC (resp. specimen 4L, and 5R). A total of two temporal bones (spec. 1R and 6L) were excluded from the final analysis leaving 10 included specimens. The former due to a prematurely collapsed SS after TL-S dissection. The latter due to a significant compression at the distal part of the SS on the post-TL-R CT-scan.

The presigmoid depth was defined as the distance between the porus and the medial border of the SS. This depth (mean: 25 mm, range: 15–38 mm, standard deviation [SD]: 5.8) did not statically significantly ( *p*_2 =_ 0.052) change after retraction of the SS during TL-R when compared to TL-S. The area of the presigmoid dura, which is approximated by constructing a trapezoid with a medial base between the jugular bulb and the tegmen, a lateral base medial to the vertical part of the SS, and the presigmoid depth, ranged from 283 to 776 mm ^2^ (mean: 413 mm ^2^ , SD: 151).

### Surgical Freedom

**Fig. 5:**
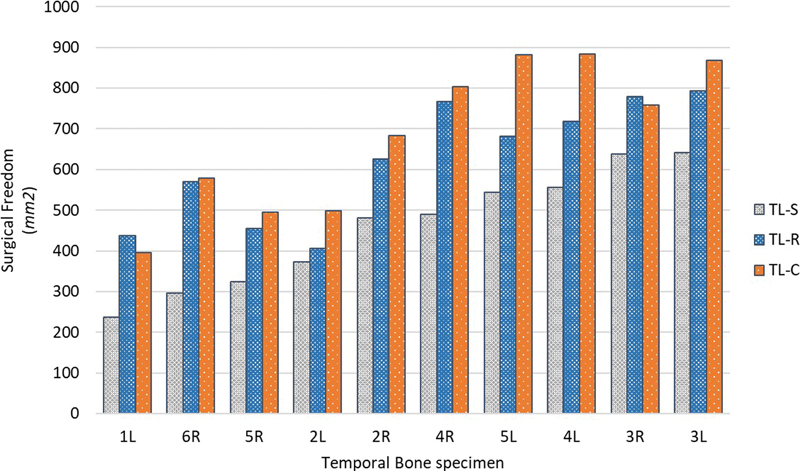
Surgical freedom (mm ^2^ ) stratified by procedure and specimen, and ranked by TL-S. TL-C, translabyrinthine with collapsed sinus; TL-R, translabyrinthine with retracted patent sinus; TL-S, translabyrinthine without sinus retraction.

**Fig. 6:**
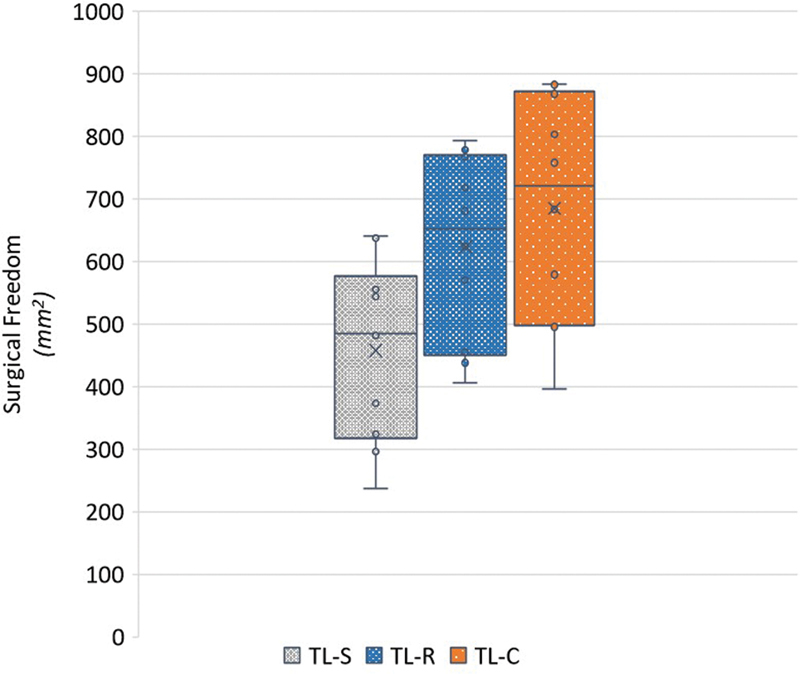
Boxplot of surgical freedom (mm ^2^ ) stratified by procedure. TL-C, translabyrinthine with collapsed sinus; TL-R, translabyrinthine with retracted patent sinus; TL-S, translabyrinthine without sinus retraction.

When retraction of the SS was performed during TL-R, SF increased by a mean of 41% (range: 9–92%, SD: 28) when compared to no retraction (TL-S). Collapsing the SS in TL-C provided a mean increase of 52% (range: 19–95%, SD: 22) compared to TL-S. In some specimens, the TL-R provided a greater SF than the TL-C; however, on average an increase of 10% (range: −10 to 30%, SD: 12) in favor of the TL-C is observed when compared to TL-R. Fig. 5 provides the SF stratified by specimen. See Fig. 6 for the spread and mean of provided SF stratified by procedure. A statistically significant difference in mean SF between both TL-R (165 ± 72 mm ^2^ , *p*_1_ < 0.01) or TL-C (227 ± 84 mm ^2^ , *p*_1_ < 0.01) and TL-S was observed. Also, the difference in mean SF between TL-C and TL-R (62 ± 77 mm ^2^ , *p*_2_ = 0.03) was statistically significant.

Of the triangles used to calculate SF, the areas of triangles A and D, projecting respectively superiorly and inferiorly above the (proximal) IAC, were most affected by the retraction with a mean increased percentage of 55 and 39%, respectively. Triangles B and C increased on average 24 and 27%, respectively.

Pearson correlation analysis of the presigmoid area and SF after TL-S showed a high positive correlation ( *r* = 0.67, *p*_2_ = 0.033). Furthermore, both presigmoid area ( *r* = −0.64, *p*_2_ = 0.047) and presigmoid depth ( *r* = −0.70, *p*_2_ = 0.024) showed a high negative correlation with the percentage of increased SF when comparing TL-C with TL-S. No correlation between TL-R SF and presigmoid area or depth was statistically significant.

### Exposure Angle

The mean EA above the IAC statistically significantly increased in both the TL-R (14 ± 11°, *p*_1_ = 0.001) and TL-C (13 ± 8°, *p*_1_ < 0.001) when compared to TL-S. The mean EA above the jugular bulb statistically significantly increased in both the TL-R (15 ± 16°, *p*_1_ = 0.006) and TL-C (14 ± 10°, *p*_1_ = 0.001) when compared to TL-S. There was no statistically significant difference in the EAs when comparing TL-C to TL-R at both the level of the IAC (−0.5 ± 6°, *p*_2_ = 0.82) and the jugular bulb (−2 ± 17°, *p*_2_ = 0.78). Both absolute EAs and the percentage increased are shown in Table 1 .

**Table 1:** Quantification of the mean exposure angle and angle of attack with various sigmoid sinus positions

Surgical approach	Exposure angle		Angle of attack
	IAC	Jugular bulb	IAC
**TL-S**	94° ± 14	78° ± 14	67° ± 13
**TL-R**	108° ± 10	93° ± 9	76° ± 13
**TL-C**	108° ± 9	91° ± 18	84° ± 13
**% increased**			
**TL-R** a	16% ± 14	25% ± 31	14% ± 8
**TL-C** a	16% ± 10	18% ± 13	26% ± 11
**TL-∆** b	0% ± 6 c	−1% ± 18 c	11% ± 9

Abbreviations: IAC, internal auditory canal; TL-C, translabyrinthine with collapsed sinus; TL-R, translabyrinthine with retracted patent sinus; TL-S, translabyrinthine without sinus retraction.

a Increased percentage of degrees compared to same specimen TL-S.

b Increased percentage of degrees of TL-C compared to same specimen TL-R.

c Statistical significancy was not reached ( *p* > 0,05).

Pearson correlation analysis of the EA in either TL-S, TL-R, or TL-C, and pre-sigmoid area or depth did not provide significant correlations.

### Angle of Attack

The mean angle of attack increased at each successive step of the resection from 67° (range: 49–88°, SD: 13) to 76° (range: 60–103°, SD: 13), and 84° (range: 60–103°, SD: 13) in TL-S, TL-R, and TL-C, respectively. These results are shown in Table 1 . The difference in mean angle of attack in TL-R (9 ± 4°, *p*_1_ < 0.001) and TL-C (17 ± 5°, *p*_1_ < 0.001) was statically significant when compared to TL-S. The difference in mean angle of attack when comparing TL-C to TL-R was 8 ± 6° ( *p*_2_ = 0.003).

Pearson correlation analysis of the angle of attack in either TL-S, TL-R, or TL-C, and presigmoid area or depth did not provide significant correlations.

## Discussion

### Technical and Methodologic Consideration

Various methods have been used to quantify exposure ranging from stereophotographic measurements of dissected cadaveric specimens 11 15 to complete digital simulation of the procedure. 12 More recently, cadaveric dissection and variables calculated from neuro-navigation coordinates have an increased interest. 6 9 13 16 29 Similarly, our technique used real-world dissection and a digital representation of this resection cavity to quantify the exposure. Creating a high-resolution 3D model of this cavity visualized and quantified the outcomes in a reproducible manner. Furthermore, it allowed for measurement and representation of angles and distances which would not easily be visually created thus far. Because of this, a strength of this study is the multimodal contribution and addition of multiple outcome measures to visualize surgical exposure.

The described method has a significant constraint in that only bony structures could be visualized on cone beam CT-scan. For this reason, it is recommended to leave a thin bony covering on areas of interests. The measurement error as a result of the thickness of this covering is, we believe, not troublesome and might even improve reproducibility since it reduces the inherent mobility soft tissue has and provides a recognizable surface across the procedures. Nevertheless, this limited mobility might have constrained the SS during retraction. Similarly, the presigmoid intact dura, when compared to a situation in which the dura is opened to access the CPA, probably constrained the movement of the SS. This hypothesis is supported by the lack of change in presigmoid depth between TL-S and TL-R. Therefore, the quantified exposure after TL-R could be an underestimation of the intraoperative attainable exposure.

Furthermore, a cadaveric model does not replicate the anatomical distortion and changes in tissue characteristics caused by CPA pathology. 6 Although the use of fresh frozen instead of formaldehyde-fixated specimens provided a more accurate representation of tissue handling, the mobility and the lack of intracranial pressure will have influenced the outcome.

The position of the SS during TL-C and the performed TL in patients can differ in various amounts. In this study, it is assumed that the collapsed position of the sinus remains stationary. However, based on the surgeon's preference, various amounts of retro-mobilization of the collapsed SS might be performed. 27 This partially compresses, but not as much as during a retrosigmoid approach, the cerebellum providing an even greater exposure than our TL-C suggested. In doing so, the difference between TL-S and TL-C increases. However, greater retrosigmoid dissection than is provided in these specimens might be required. It is plausible that the surgical exposure in TL-R will similarly increase with greater retrosigmoid dissection with the advantage of a more protected (by bony covering) SS decreasing the risk of lesions or postoperative sinus thrombosis.

### Quantification of Surgical Exposure

Although the surgical exposure seemed to increase the most with the collapsed SS (TL-C), retracting a skeletonized SS conserves patency in exchange for approximately 10% of SF and 8 degrees in the angle of attack; there seemed to be no statistically significant difference in EAs over the IAC and jugular bulb.

The simultaneous changed angle of attack in combination with the unchanged EAs seems contradictory. One hypothesis for this phenomenon could be that the effect size is smaller than anticipated, since the position of the sinus was fixed to the porus by the presigmoid dura. Thus, the mobility of the sinus is not (merely) a posterior translation, but also medial translation, rotating around the porus by its dural-tether. This motion would alter the shearing lines required for EA measurement less than a pure posterior translation. During an inpatient operative procedure, this dura is incised ruling out this medial translation, therefore possibly increasing EA. Another hypothesis could be the assumed change in shape of the SS lumen during the TL-C: from a mostly round shape in TL-S and TL-R to a flat shape in line with the dura. This change in shape would impact the anterior border of the SS, relevant to angle of attack, more than the medial and lateral borders, which are relevant to the approach angle. A third explanation could be that, because the intersection of the two tangential lines in EA is relatively close to the SS itself, the small measurement error is amplified.

During actual resection of a large tumor, one does not constrict oneself to the transverse plane, such as our measurement of angle of attack for the sake of consistency. It is for instance easier to reach the lower cranial nerves when approaching from the sinodural angle, while the trigeminal nerve can be better reached from the inferior side of the craniotomy. While the AA will differ in absolute degrees based on the chosen superior-inferior starting position, we expect that the relative differences between AA in the various procedures will remain comparable regardless of the superior-inferior starting position.

The large spread of provided exposures across the specimens would normally be considered as negative in quantitative anatomical studies; however, since this study sequentially performed all procedures on each specimen, this might be considered as one of its strengths; it evaluated specimens with limited SF ranging from 250 mm ^2^ up to a generous 650 mm ^2^ after TL-S. The presigmoid area was significantly correlated to the SF post-TL-S. Furthermore, an inverse correlation was observed with the percentage increased SF after collapsing the sinus. In other words, the greater the distance between the porus and the sinus, the less ancillary exposure is gained by collapsing the sinus. Thus, retracting a skeletonized sinus might in those cases result in satisfactory exposure. This claim is more visualized by the boxplot showing the close relationship between the SF of TL-R and TL-S, and the fact that the top 40% of TL-R SF is greater than the bottom 50% of TL-C.

### Practical Considerations, Clinical Implications, and Future Perspectives

A limitation of using this model is the absence of venous and dural (CSF) pressure which would aid in keeping the SS patent. The overall aim of retracting a skeletonized sinus was to ensure patency. During this study patency was violated in one specimen during the retraction. To mobilize the sinus, careful dissection of the peri-sinusoid bone is primordial. During the retraction, depending on the specific thickness of the skeletonized bone, some fractioning of this bone might appear. To minimize this probability, a relatively conservative skeletonization should be aimed for, leaving ample bone on the sinus. This is especially important on the anterolateral walls of the sinus to benefit from the Roman bridge effect caused by the rounded shape during retraction, and distally near the jugular bulb since this part seemed to be the least compliant to this direction of motion.

Our study showed the feasibility and expected SF of this maneuver in a human cadaveric head. Based on our result, further research in development of this technique seems promising if SS-associated morbidity could be reduced. If effective, it might, amongst others, reduce the relative risk of sinus thrombosis after TL resection to a value similar to retrosigmoid sections in which the SS remains undisturbed; thus reducing the odds by a factor of 19.82 (95% confidence interval [CI] = 1.75–224, *p* = 0.007). 30 Although the clinical course of sinus thrombosis is often relatively benign, it significantly increases the odds of CFS leak (odds ratio [OR]: 3.197; 95% CI: 1.899–5.382; *p* < 0.001) and CFS dynamic alteration (OR: 3.625; 95% CI: 2.370–5.543; *p* < 0.001). 31 Any reduction in exposure might theoretically increase risk and decrease the ability to resolve intra-operative issues. Whether this reduction in morbidity justifies a slightly reduced exposure is still open for debate and dependent on the surgeons' prior experiences and preferences. Further optimization of the technique, involving amongst others the ideal extent of retrosigmoid bone removal, should occur. Moreover, the ability to perform a combined pre- and retrosigmoid approach in which the sinus is protected might be advantageous. Prior to in-human application of this maneuver to quantify the reduction in morbidity, we believe that further research in a model evoking venous and dural pressures should be performed to quantify the effect on the blood flow in these sinuses. Furthermore, these models would allow for more accurate determination of the expected exposure during TL-C, which was a limitation in our current model.

Augmented and mixed reality (AR and MR) applied to neuro- and skull base surgery has become a promising research interest. Fick et al showed the reliability, accuracy, and speed of AR in clinical neurosurgical practice and its ability to fully automatic segment tumors. 32 Pennacchietti et al demonstrated the additional benefit of using AR in endoscopic-assisted skull base surgery. 7 The benefits of this technology reaches beyond education and preoperative planning, and can provide simultaneous multisource information to even experienced surgeons. 33 Likewise, MR, in which the information is not only projected over the object, but also provides interactive virtual data, could in the future be helpful in estimating how exposure will change with a dynamic mobilization of the soft tissue anatomy. Studies like this one, which quantify not only static exposure, but also the compliance of structures, could be valuable to these developments. Ideally, the operating team could model and visualize in 3D the expected exposure in the CPA through the petrous bone using various retractions of the SS using patient-specific data. Further research in this new field of quantitative dynamic anatomy is necessary.

In conclusion, while in most cases the exposure is optimized with a collapsed SS, in nearly half of the cases exposure after retracted SSs was at least equal to that of the collapsed sinuses. With patient selection, a TL resection in which the skeletonized SS is retracted provides sufficient exposure for adequate and safe tumor resection.
